# Movement-Related Activity of Human Subthalamic Neurons during a Reach-to-Grasp Task

**DOI:** 10.3389/fnhum.2017.00436

**Published:** 2017-09-07

**Authors:** Monika Pötter-Nerger, Rene Reese, Frank Steigerwald, Jan Arne Heiden, Jan Herzog, Christian K. E. Moll, Wolfgang Hamel, Uri Ramirez-Pasos, Daniela Falk, Maximilian Mehdorn, Christian Gerloff, Günther Deuschl, Jens Volkmann

**Affiliations:** ^1^Department of Neurology, Christian-Albrechts-University Kiel, Germany; ^2^Department of Neurology, University Hamburg-Eppendorf Hamburg, Germany; ^3^Department of Neurology, University Rostock Rostock, Germany; ^4^Department of Neurology, Julius-Maximilian University Würzburg, Germany; ^5^Department of Neurophysiology, University Hamburg-Eppendorf Hamburg, Germany; ^6^Department of Neurosurgery, University Hamburg-Eppendorf Hamburg, Germany; ^7^Department of Neurosurgery, Christian-Albrechts-University Kiel, Germany

**Keywords:** subthalamic nucleus, deep brain stimulation, Parkinson’s disease, neurophysiology, beta oscillation, reach-to-grasp movement

## Abstract

The aim of the study was to record movement-related single unit activity (SUA) in the human subthalamic nucleus (STN) during a standardized motor task of the upper limb. We performed microrecordings from the motor region of the human STN and registered kinematic data in 12 patients with Parkinson’s disease (PD) undergoing deep brain stimulation surgery (seven women, mean age 62.0 ± 4.7 years) while they intraoperatively performed visually cued reach-to-grasp movements using a grip device. SUA was analyzed offline in relation to different aspects of the movement (attention, start of the movement, movement velocity, button press) in terms of firing frequency, firing pattern, and oscillation. During the reach-to-grasp movement, 75/114 isolated subthalamic neurons exhibited movement-related activity changes. The largest proportion of single units showed modulation of firing frequency during several phases of the reach and grasp (polymodal neurons, 45/114), particularly an increase of firing rate during the reaching phase of the movement, which often correlated with movement velocity. The firing pattern (bursting, irregular, or tonic) remained unchanged during movement compared to rest. Oscillatory single unit firing activity (predominantly in the theta and beta frequency) decreased with movement onset, irrespective of oscillation frequency. This study shows for the first time specific, task-related, SUA changes during the reach-to-grasp movement in humans.

## Introduction

Several studies have attempted to elucidate the role of the basal ganglia in motor control.

An important contribution to the understanding of basal ganglia function was supplied by animal studies. Single cell recordings in healthy primates during visually triggered arm movements revealed phasic, movement-related activity changes in the globus pallidus pars internum (GPi), putamen, nucleus caudatus and subthalamic nucleus (STN) (Georgopoulos et al., 1983; Jaeger et al., 1995; Lebedev and Nelson, 1999). Movement-associated activity changes were observed in the primary motor cortex and supplementary motor area (Alexander and Crutcher, 1990a), and rhythmic oscillatory activity changes in particular were found in the somatosensory (Lebedev and Nelson, 1995) and premotor cortex (Lebedev and Wise, 2000). Movement-related cells were somatotopically arranged in segregated, parallel motor loops (DeLong et al., 1984a; Wichmann et al., 1994a). Special interest has focused on the function of the STN as a central key station in movement control, since alterations of its neuronal cell discharges were associated with behavioral changes (Bergman et al., 1994; Wichmann et al., 1994a,b).

More recently, routine use of deep brain stimulation in Parkinson’s disease (PD) has enabled direct, single unit recordings from the STN during the surgical placement of the stimulating electrodes. To date, most attempts at characterizing human STN single unit activity (SUA) were focused on the resting state. When lying immobile, PD patients demonstrated an increased mean firing rate and a relatively larger fraction of STN neurons exhibiting burst-like, oscillatory and synchronized activity, predominantly in the beta frequency (Hutchison et al., 1998; Magariños-Ascone et al., 2000) compared to recordings in essential tremor patients (Steigerwald et al., 2008).

Few studies have investigated single cell activity during voluntary movements in humans. Movement-related changes of STN unit activity have been described during self-paced, unconstrained movements of the upper and lower limb, such as simple extension-flexion movements (Magariños-Ascone et al., 2000; Rodriguez-Oroz et al., 2001; Abosch et al., 2002), during visually guided joystick movements (Amirnovin et al., 2004; Gale et al., 2009), voluntary, repetitive chest-to-target reaching movements (Levy et al., 2002), or during cursor-tracking tasks (Hanson et al., 2012). Movement-related cells were distributed in a somatotopic fashion within the human STN (Theodosopoulos et al., 2003; Romanelli et al., 2004, 2005). Although these studies revealed the existence of movement-related activity changes of the STN, they did not relate activity changes to temporal features and kinematic aspects of more complex, everyday motor tasks.

Reach-to-grasp movements represent functionally relevant movements in the daily routine, which recently gained new interest for decoding movement-related neuronal activity to improve the design of neural prosthetics (Aflalo et al., 2015; Pruszynski and Diedrichsen, 2015). Goal-directed movements and hand-shaping were intensively studied by recording cortical activity within the human posterior parietal area (Aflalo et al., 2015; Klaes et al., 2015), primate anterior intraparietal and premotor area (Lehmann and Scherberger, 2015; Michaels et al., 2015), and by functional MRI investigations of the visuomotor cortical areas in man (Gutteling et al., 2015). The reach-to-grasp movement requires the coordination of two different subcomponents: the “reach”, which is mainly executed by proximal muscles of the upper limb reaching the hand towards the goal, and the “grasp”, exerted by distal hand muscles (Jeannerod, 1984). These components are assumed to be encoded by separate brain circuits: the dorsomedial loop involving the superior parietal and dorsal premotor cortex, which is active during all phases of the movement (Fattori et al., 2010), and the dorsolateral circuit including the inferior parietal and ventral premotor cortex governing predominantly the grasp formation (Cavina-Pratesi et al., 2010), which are differentially activated depending on extrinsic and intrinsic object properties (Monaco et al., 2015). Animal data suggest additional involvement of subcortical structures such as the red nucleus (van Kan and McCurdy, 2002), GPi (Wenger et al., 1999) and cerebellum (Mason et al., 2006).

Kinematic analyses in untreated PD patients has revealed deterioration of several aspects of the reach-to-grasp task, such as decreased velocity during the reaching phase and a disturbed timing pattern of the coordination of the subcomponents of the movement (Alberts et al., 2000). In a preceding study, we observed that high-frequency stimulation in the STN improved distinct aspects of the reach-to-grasp task, in particular the maximum velocity and movement time (MT) during the reaching period, but had little impact on the grip formation (Pötter-Nerger et al., 2013).

The goal of this study was to investigate the role of the STN during the combined reach-to-grasp task by performing intraoperative microrecordings during motor execution. We aimed to relate STN single cell activity to certain aspects of the reaching phase and grip formation, and evaluate the impact of the movement on neuronal firing frequency, oscillation and synchronization.

## Materials and Methods

Twelve PD patients (seven women, mean age 62.0 ± 4.7 [SD] years) were enrolled after providing written informed consent. This study was carried out in accordance with the recommendations of local ethics committee of Christian-Albrechts-University Kiel with written informed consent from all subjects. All subjects gave written informed consent in accordance with the Declaration of Helsinki. The protocol was approved by the local ethics committee of Christian-Albrechts-University. The decision to perform surgery was not influenced by participation within this study. Detailed information of patient demographics and clinical characteristics are provided in Table 1.

**Table 1:** Clinical data of Parkinsonian patients.

Patient	Gender	Age (years)	Disease duration (years)	LEDD preoperative (mg)	LEDD postoperative (mg)	Total UPDRS III preoperative MED OFF	Total UPDRS III preoperative MED ON	Total UPDRS III postoperative STIM ON MED OFF	DBS stimulation parameter postoperative
01	Female	58	8	1380	450	49	30	22	K1 CASE + C1-, 2.4 V, 60 μs, 130 Hz
									K2 CASE + C5-, 2.7 V, 60 μs, 130 Hz
02	Female	58	18	1182	450	45	27	31	K1 CASE + C1-, 2.9 V, 60 μs, 130 Hz
									K2 CASE + C6-, 2.8 V, 60 μs, 130 Hz
03	Male	65	7	1331	715	23	11	9	K1 CASE + C1-, 2.5 V, 60 μs, 130 Hz
									K2 CASE + C5-, 2.5 V, 60 μs, 130 Hz
04	Male	58	13	1226	750	31	20	18	K1 CASE + C2-, 3.9 V, 60 μs, 130 Hz
									K2 CASE + C5-, 4.2 V, 60 μs, 130 Hz
05	Female	72	16	1325	300	32	15	23	K1 CASE + C1–3-, 1.5 V, 60 μs, 180 Hz
									K2 CASE + C5–6-, 3.6 V, 60 μs, 180 Hz
06	Male	62	21	1877	600	33	17	16	K1 CASE + C1-, 3.1 V, 60 μs, 180 Hz
									K2 CASE + C5-, 3.0 V, 60 μs, 180 Hz
07	Female	69	10	850	350	38	22	14	K1 CASE + C1-, 3.4 V, 60 μs, 130 Hz
									K2 CASE + C5-, 3.2 V, 60 μs, 130 Hz
08	Male	60	4	719	150	39	14	11	K1 CASE + C1-, 2.9 V, 60 μs, 130 Hz
									K2 CASE + C5-, 2.9 V, 60 μs, 130 Hz
09	Female	57	25	840	260	36	4	22	K1 CASE + C2-, 3.7 V, 60 μs, 180 Hz
									K2 CASE + C5-, 2.8 V, 60 μs, 180 Hz
10	Female	60	10	1033	225	53	13	13	K1 CASE + C1-, 3.7 V, 60 μs, 210 Hz
									K2 CASE + C5-, 4.0 V, 60 μs, 210 Hz
11	Male	62	4	250	200	49	26	10	K1 CASE + C1-, 2.0 V, 60 μs, 180 Hz
									K2 CASE + C5–6-, 3.1 V, 60 μs, 180 Hz
12	Female	63	10	1191	375	36	7	11	K1 CASE + C1-, 3.1 V, 60 μs, 130 Hz
									K2 CASE + C4-, 4.2 V, 60 μs, 130 Hz
mean ± SD	7 female, 5 male	62.0 ± 4.7	12.2 ± 6.6	1100.3 ± 406.7	402.1 ± 198.3	38.7 ± 8.8	17.2 ± 8.1	16.7 ± 6.7	K1 2.9 ± 0.7 V, 60 ± 0 μs, 153.3 ± 29.9 Hz
									K2 3.3 ± 0.6 V, 60 ± 0 μs, 153.3 ± 29.9 Hz

*The table summarizes the clinical data of the examined parkinsonian patients preoperatively and postoperatively at the first follow-up examination. LEDD, L-dopa daily equivalent dosage. Total UPDRS III: sum score of the complete motor part of the Unified Parkinson’s Disease Rating Scale. MED OFF: without antiparkinsonian medication. MED ON: after suprathreshold administration of soluble L-dopa medication (morning dosage × 1.5). STIM ON MED OFF: postoperatively without medication with stimulation switched on. DBS, deep brain stimulation. K1: electrode in the right subthalamic nucleus. K2: electrode in the left subthalamic nucleus. C: contact of the DBS electrode, C 0 and C 4 representing the most ventral electrode contacts, C 3 and C 7 representing the most dorsal electrode contacts. Mean and standard deviation of the mean of all patients are given in the last row. SD, standard deviation*.

### Experimental Procedure

The day before surgery, the patients were familiarized with the motor task by performing 1–2 × 20 trials of the reach-to-grasp task outside the operating theatre.

For surgery, antiparkinsonian medication was withdrawn to allow a stable off-state (dopamine agonists at least 72 h, levodopa at least overnight). The optimal target site for electrode implantation was determined by multi-trajectory microelectrode recordings (BenGun, five microelectrodes, recordings in steps of 0.1–1 mm) and clinical evaluation of macrostimulation responses as described previously (Steigerwald et al., 2005, 2008). Briefly, up to five stainless microelectrodes (FHC, Bowdoinham, ME, USA) were simultaneously advanced in steps of 0.5 mm. Amplification, visual display, and audio monitoring of the signal were handled by the Leadpoint microrecording system (Medtronic, Minneapolis, MN, USA). The analog output of this system was fed into a second-stage biosignal amplifier (TPM, Luneburg, Germany) for band-pass filtering (0.3–10 kHz, gain of 100). Recordings were digitized using the CED 1401 system (sampling rate of 25 kHz, Cambridge Electronic Design [CED], Cambridge, UK) and stored for offline analysis on a personal computer.

**Figure 1:**
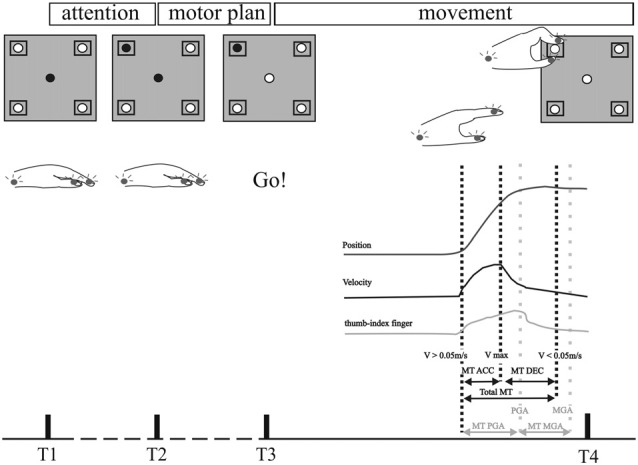
Experimental set up and data analysis of kinematic data. Schematic outline of the motor tasks. The board (gray squares, upper row) carried a central light-emitting diode (LED) and four cubic grip objects (0.02 m × 0.045 m × 0.085 m) with response buttons and target LEDs. The central light was illuminated for 3 s as a warning cue at the start of each trial. Illumination of the target LED and dimming of the central LED was the starting cue for each movement. A successful button press was acknowledged by turning off the target LED. Patients performed the reach-to-grasp task, including a reaching phase and grip formation around the response button. The movement was analyzed with recorded kinematic data of Zebris system. Upper row: position curve of the hand wrist marker in the sagittal plane. Middle row: velocity curve of the hand wrist marker. Lower row: distance between the two markers on the thumb and index finger. Black dotted lines represent the time points in the velocity curve displaying the beginning of the movement (when velocity exceeds 0.05 m/s), maximum velocity and the end of the movement (when velocity falls below 0.05 m/s). Gray dotted lines represent the time points of the maximal peak distance between thumb and index finger (PGA) and minimal distance (MGA). Movement times (MT) were calculated from these discrete time points as demonstrated above. MT ACC: acceleration time (time from the beginning of the movement to maximal velocity). MT DEC: deceleration time (time from maximal velocity to the end of the movement). Total MT: duration of the whole movement (calculated from the velocity curve from the beginning to the end of the movement). MT PGA: time to peak grip aperture (from the beginning of the movement to the time of maximal thumb-index finger distance). MT MGA: grip closure time (time from maximal to minimal distance of thumb and index finger). Reproduced with permission from Pötter-Nerger et al. (2013).

During intraoperative neurophysiological testing, the reach-to-grasp task was additionally performed at each recording site inside the entire left STN, where spike activity with sufficient signal-noise ratio was encountered on at least one electrode. The experiment prolonged the surgery by approximately 30 min. The entire neurophysiological mapping was performed under local anesthesia without any sedatives. The subjects were in a supine position on the operating table, with the head fixed in a stereotactic frame. A response panel was placed within reach distance and within their visual field. The right hand had to be placed palm down beside the body on the operating table, as the resting condition between reaching movements. Details of the response board, the reach-to-grasp tasks, kinematic recordings with the ultrasound movement analysis system (CMS 70 P4-V5, Zebris, Germany), and electromyographic (EMG) recordings (right deltoid and first dorsal interosseus muscles) have been outlined previously (Pötter-Nerger et al., 2013) and are illustrated in Figure 1. Briefly, small ultrasound emitting markers were fixed to the tip of the thumb and index finger, the radial styloid process, the lateral epicondyle of the elbow, and the acromion of the shoulder. A recording panel of three microphones measured the marker movements in 3D space, thus providing information about kinematic characteristics of the reach-to-grasp movement. A central light-emitting diode (LED) on the response board was illuminated as a warning cue, thereafter a directional cue was provided by an LED on the response cylinder, and the central LED switched off as a GO cue with a variable delay. After the GO cue, subjects were instructed to perform the movement towards the response cylinder quickly but carefully. The sequence was repeated 10 times with an intertrial interval of at least 10 s (randomized intervals 10–20 s).

### Data Analysis

#### Spike Sorting

Microrecordings were analyzed offline using the Spike 2 software version 5 (CED, Cambridge, UK) and Neuroexplorer version 4 (NEX Technologies, Littleton, MA, USA), as described previously (Steigerwald et al., 2005, 2008). SUA was discriminated by threshold spike detection, template matching (70% maximum percent amplitude match, template focused on the initial negatively slope of the spike), controlled by cluster analysis with principal component analysis and final visual inspection.

#### Investigation of Movement-Related STN Activity with Peristimulus Time Histograms

Movement-related modulation of SUA was investigated by calculating peristimulus time-histograms (PSTH) on certain kinematic aspects of the reach-to-grasp movement. Reference events for the PSTH were the time stamps for the warning and GO cue, as well as reference events extracted from kinematic Zebris measures ((1) start of the hand-reaching phase, when wrist sensor velocity exceeded 0.05 m/s; (2) peak velocity of the wrist sensor; (3) end of the reaching period, when wrist sensor velocity fell below 0.05 m/s; (4) time of maximal grip aperture, as indicated by maximal distance between thumb and index finger marker; (5) time of minimal distance between thumb and index finger during button press), and reference events extracted from EMG activity (onset of the deltoid and first dorsal interosseus muscle activity). The onset of EMG activity was determined after DC offset correction, rectification and smoothing (time constant 0.1 s), with a threshold detection algorithm (threshold 0.04 V) and final visual inspection.

PSTH (bin 0.025 s in post-processing smoothing of PSTH with boxcar filter 3 bins, unit spikes per second) were calculated in NEX with two different time ranges around the event (−0.5 s to 3 s around the digital GO signal to obtain an overview of SUA activity during the entire course of the movement and −0.5 s to 1.5 s for each reference event for detailed SUA modulation). Peaks in PSTH were considered significant when they exceeded the 95% confidence interval around the mean expected firing rate of the whole recording.

#### Correlation of STN-SUA with Reach Velocity

SUAs with a PSTH demonstrating modulation during the reaching phase of the reach-to-grasp movement were correlated with the velocity of the wrist sensor by breaking the spike train and velocity curve into time segments of 0.1 s bins. Two-sided Pearson testing was performed at a significance level of *p* < 0.05.

#### Characterization of Neuronal Firing Pattern by Interspike-Interval Histograms

The firing pattern of SUA was categorized as described previously (Hassani et al., 1996; Steigerwald et al., 2008). The firing pattern of each neuron was predetermined based on the visual inspection of the spike train and the characteristics of the interspike-interval histogram (ISIH). ISIH (interval 0–0.25 s, bin 0.001 s, smoothed with filter 3 bins) were calculated for SUA at rest (−3 s to 0 s prior to attentional signal) and during movement (0–3 s after GO signal). ISIH were classified into three types according to their asymmetry index (AI), calculated by the ratio of mode ISI/mean ISI and the coefficient of the variance (CV): (1) bursting or burst-like firing pattern (AI < 0.55; CV < 1.25); (2) irregular firing pattern (AI < 0.55; CV > 1.25); (3) regular tonic firing pattern (AI close to unity, at least >0.55; CV < 1). To assess possible differences in firing characteristics at rest and during movement, we compared mean frequencies and mean CV as a marker of firing regularity by paired *t*-tests (level of significance *p* < 0.05). In addition, we investigated whether the firing pattern of a neuron at rest was associated with the pattern during movement by performing two separate Huynh-Feldt corrected two-way ANOVAs with the factors neuron ((1) facilitation vs. inhibition; (2) reaching vs. grip vs. post-movement vs. polymodal vs. no reaction) and condition (rest vs. movement).

#### Characterization of Neuronal Firing Patterns by Autocorrelation and Power Spectral Density Analysis

Autocorrelation functions (1000 ms, bin size 1 ms, offset 500 ms) were calculated for each SUA at rest (SUA activity outside of the time range from the warning signal and the end of the movement) and during the prehensile movement (SUA activity within the time range from the GO signal to the end of the movement) and analyzed by the method of Raz et al. (1996); (Amirnovin et al., 2004) to detect significant oscillations. For the autocorrelation, the trough of the refractory period ± around the time zero was removed first to reduce the high-frequency noise of the low pass filtered (100 Hz) signal. Consecutive power spectra (1.953 Hz resolution, hamming window) were analyzed for peaks between 1 Hz and 100 Hz exceeding five standard deviations above the mean power, and normalized by scaling to RMS. The oscillatory activity was classified according to the peak frequency of the power spectrum into extended theta (1–7 Hz), alpha (8–12 Hz), beta (13–35 Hz), lower gamma (36–60 Hz), or higher gamma activity (60–100 Hz).

To analyze differences of the mean power of the autocorrelogram between rest and movement, 2-factorial, Huynh-Feldt-corrected ANOVA with the factors condition (rest vs. movement) and the two main frequencies (theta vs. beta frequency) was conducted.

Results are presented as mean ± standard deviation, unless otherwise stated.

## Results

We were able to extract the activity of 114 SUA during reach-to-grasp movements in 12 patients. These SUA were examined in terms of their movement-related activity, their firing characteristics and their location within the STN.

### Kinematic Aspects of Intraoperative Reach-to-Grasp Task

The kinematics of the intraoperatively-performed reach-to-grasp movements (Table 2) were comparable to the movement performance of PD patients observed postoperatively in the STIM OFF condition in a previously described larger cohort (Pötter-Nerger et al., 2013). The total MT was slightly prolonged compared to controls in literature (1.22 ± 0.23 s). During the reaching phase, the acceleration phase (0.44 ± 0.14 s of total MT) was usually shorter than the deceleration phase (0.77 ± 0.18 s), the peak velocity (0.25 ± 0.08 m/s) was achieved at 36.75 ± 8.58% of the total MT. The grip formation for the button press developed in parallel to the reaching phase, with an initial opening of the grip aperture between thumb and index finger (maximal distance 65.82 ± 10.53 mm) followed by the grip closure (minimal distance 45.67 ± 9.03 mm) during the button press. The time to maximal grip aperture was 0.86 ± 0.27 s at 70.76 ± 4.26% of the total MT, and thus occurred during the deceleration phase of the reaching phase near to the end of the movement.

**Table 2:** Kinematic characteristics of reach-to-grasp movement.

	Intraoperative MED OFF	Postoperative (subgroup patients) STIM OFF MED OFF	Postoperative (subgroup patients) STIM ON MED OFF
	Mean ± SD	Mean ± SD	Mean ± SD
**Reach-to-grasp movement**			
V max (m/s)	0.25 ± 0.08	0.29 ± 0.13	0.33 ± 0.11
Total MT (s)	1.22 ± 0.23	1.25 ± 0.41	1.07 ± 0.36
MT ACC (%)	36.75 ± 8.58	36.89 ± 3.77	39.09 ± 4.09
MT DEC (%)	63.25 ± 8.58	63.11 ± 3.77	60.91 ± 4.09
MT PGA (%)	70.76 ± 4.26	74.13 ± 7.03	68.50 ± 3.63
Distance PGA-MGA (mm)	20.14 ± 7.96	19.90 ± 0.80	25.61 ± 5.36

*The table demonstrates mean and standard deviation (SD) of the kinematic parameters of the reach-to-grasp movement in all 12 patients tested intraoperatively (first column) and in a subgroup of three Parkinson’s disease patients who were tested additionally postoperatively without (second column) and with subthalamic stimulation (third column). MED OFF: without antiparkinsonian medication. STIM OFF MED OFF: without deep brain stimulation and without antiparkinsonian medication. STIM ON MED OFF: with deep brain stimulation but without antiparkinsonian medication. V max (m/s): maximal velocity of the hand wrist during the reaching phase. Total MT (s): total movement time calculated from the velocity curve of the hand wrist from the beginning (*V* > 0.05 m/s) to the end (*V* < 0.05 m/s) of the reaching phase. MT ACC (%): acceleration time from the beginning of the movement (*V* > 0.05 m/s) to the time when maximal velocity is reached expressed as percentage of the total movement time. MT DEC (%): deceleration time from the time of maximal velocity to the end of the movement (*V* < 0.05 m/s), calculated as percentage of the total movement time. MT PGA (%): movement time from the beginning of the movement (*V* > 0.05 m/s) to the time when maximal peak grip aperture between thumb and index finger is reached as percentage of the total movement time. Distance PGA-MGA (mm): difference between the maximal and minimal distance between thumb and index finger during the grip formation. The movement kinematics intraoperatively were comparable to those in the postoperative OFF condition, subthalamic stimulation improved mainly the velocity during the reaching phase*.

Three of the intraoperatively-tested PD patients were also part of the cohort tested postoperatively (Pötter-Nerger et al., 2013). STN-DBS improved maximal velocity (0.33 ± 0.11 m/s), slightly widened grip aperture (maximal distance 74.79 ± 9.64 mm, minimal distance 50.09 ± 10.73 mm), but induced no changes in the velocity profile (MT acceleration 39.09 ± 4.09%; MT deceleration 60.91 ± 4.09%; time to maximal grip aperture 68.5 ± 3.63% of total MT).

### Movement-Related Activity of STN Neurons

During the reach-to-grasp movement, 75 (65.79%) of the isolated 114 STN neurons exhibited movement-associated activity changes. The activity of 56/75 neurons (74.67%) was facilitated during the movement, 17/75 neurons (22.67%) exhibited both facilitation and inhibition to different aspects of the movement, and only 2/75 neurons (2.67%) were exclusively inhibited during the movement.

**Figure 2:**
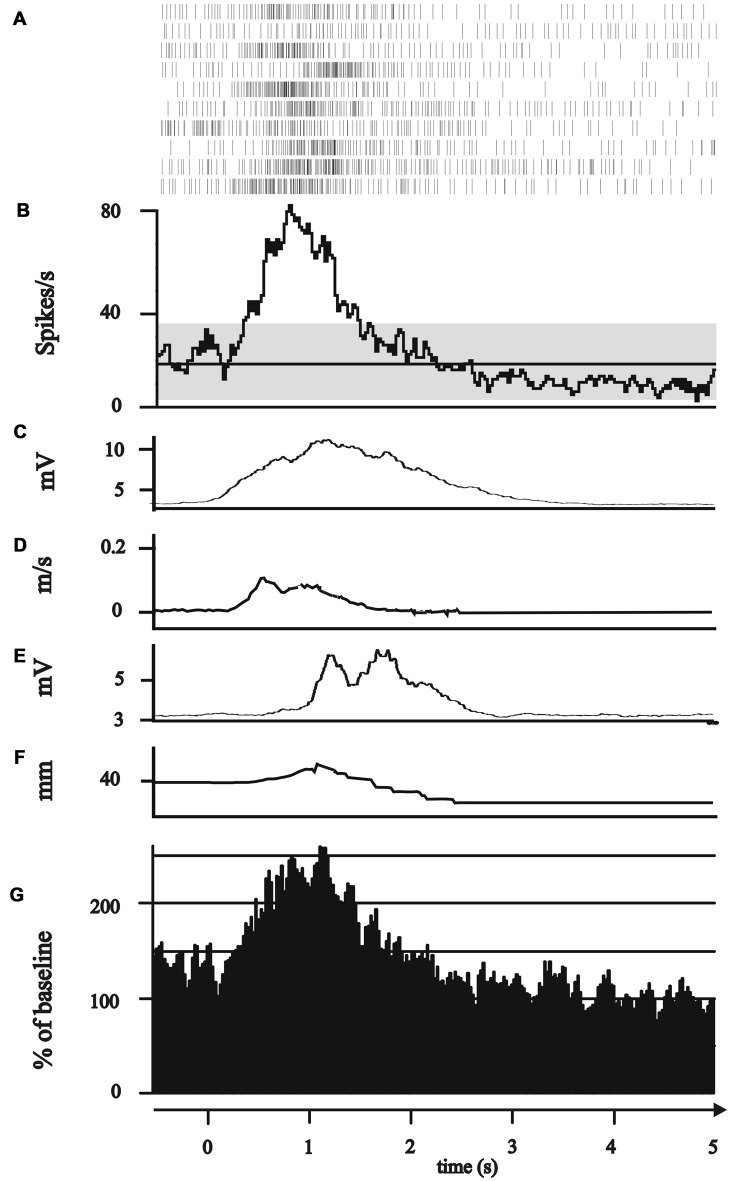
Modulation of subthalamic neuronal activity during the reaching phase. **(A–G)** All plots represent 0.5 s before to 5 s after the GO/Start signal of the movement. **(A–F)** Exemplary single neuron recorded of the posterior electrode in 1.4 mm above the target point. **(A)** Peri-event raster (bin 0.025 s of neuronal firing of the subthalamic cell during the 10 runs of reach-to-grasp movements. **(B)** Peri-event histogram of subthalamic activity (bin 0.025 s smoothed with boxcar filter of 3 bin) with confidence interval 95% (gray horizontal bar) of mean expected firing rate. **(C)** Rectified, smoothed and averaged electromyographic (EMG) activity of right deltoid muscle. **(D)** Average curve of the velocity profiles of the Zebris marker attached to the hand wrist. **(E)** Rectified, smoothed and averaged EMG of the first dorsal interosseus muscle. **(F)** Profile of the distance between thumb and index finger recorded by Zebris marker. **(G)** Grand average of all 14 reaching neurons.

**Figure 3:**
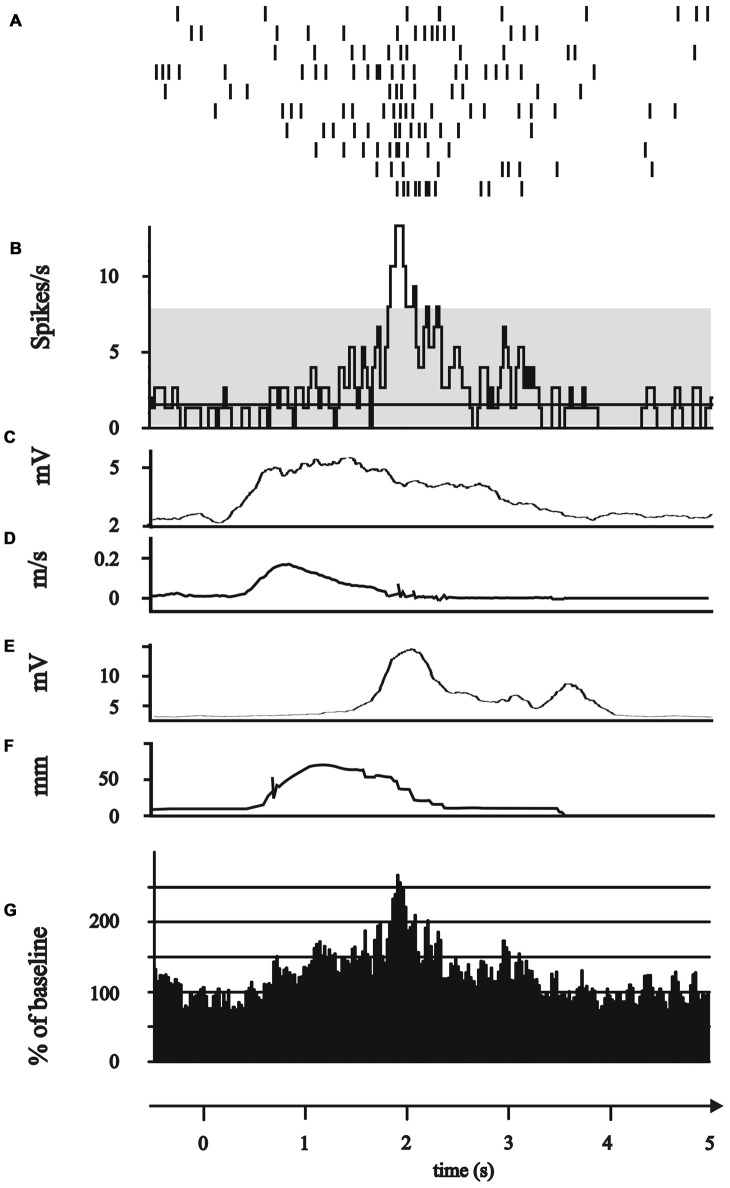
Modulation of subthalamic neuronal activity during the grip phase. **(A–G)** All plots represent 0.5 s before to 5 s after the GO/Start signal of the movement. **(A–F)** Exemplary single neuron recorded of the posterior electrode in 2 mm above the target point. **(A)** Peri-event raster (bin 0.025 s of neuronal firing of the subthalamic cell during the 10 runs of reach-to-grasp movements. **(B)** Peri-event histogram of subthalamic activity (bin 0.025 s smoothed with boxcar filter of 3 bin) with confidence interval 95% (gray horizontal bar) of mean expected firing rate. **(C)** Rectified, smoothed and averaged EMG activity of right deltoid muscle. **(D)** Average curve of the velocity profiles of the Zebris marker attached to the hand wrist. **(E)** Rectified, smoothed and averaged EMG of the first dorsal interosseus muscle. **(F)** Profile of the distance between thumb and index finger recorded by Zebris marker. **(G)** Grand average of all 10 grip neurons.

**Figure 4:**
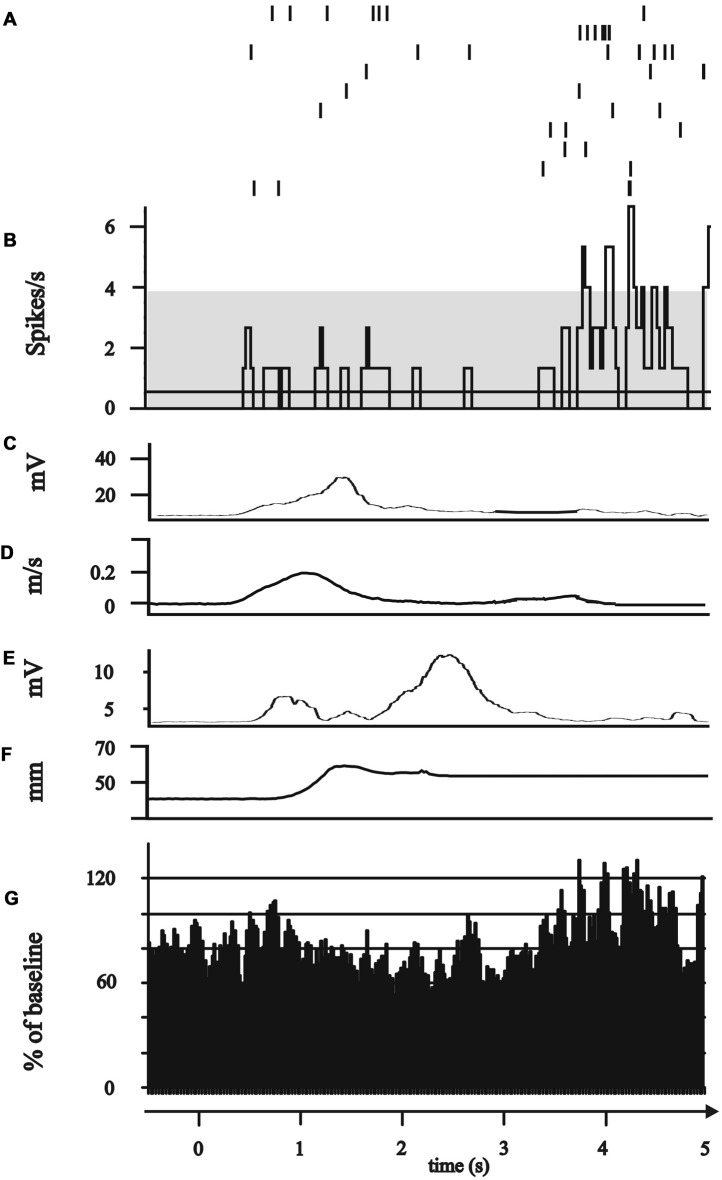
Modulation of subthalamic neuronal activity after completion of the movement (“post-movement activation”). **(A–G)** All plots represent 0.5 s before to 5 s after the GO/Start signal of the movement. **(A–F)** Exemplary single neuron recorded of the anterior electrode in 0.1 mm below the target point. **(A)** Peri-event raster (bin 0.025 s of neuronal firing of the subthalamic cell during the 10 runs of reach-to-grasp movements. **(B)** Peri-event histogram of subthalamic activity (bin 0.025 s smoothed with boxcar filter of 3 bin) with confidence interval 95% (gray horizontal bar) of mean expected firing rate. **(C)** Rectified, smoothed and averaged EMG activity of right deltoid muscle. **(D)** Average curve of the velocity profiles of the Zebris marker attached to the hand wrist. **(E)** Rectified, smoothed and averaged EMG of the first dorsal interosseus muscle. **(F)** Profile of the distance between thumb and index finger recorded by Zebris marker. **(G)** Grand average of all six post-movement activation neurons.

**Figure 5:**
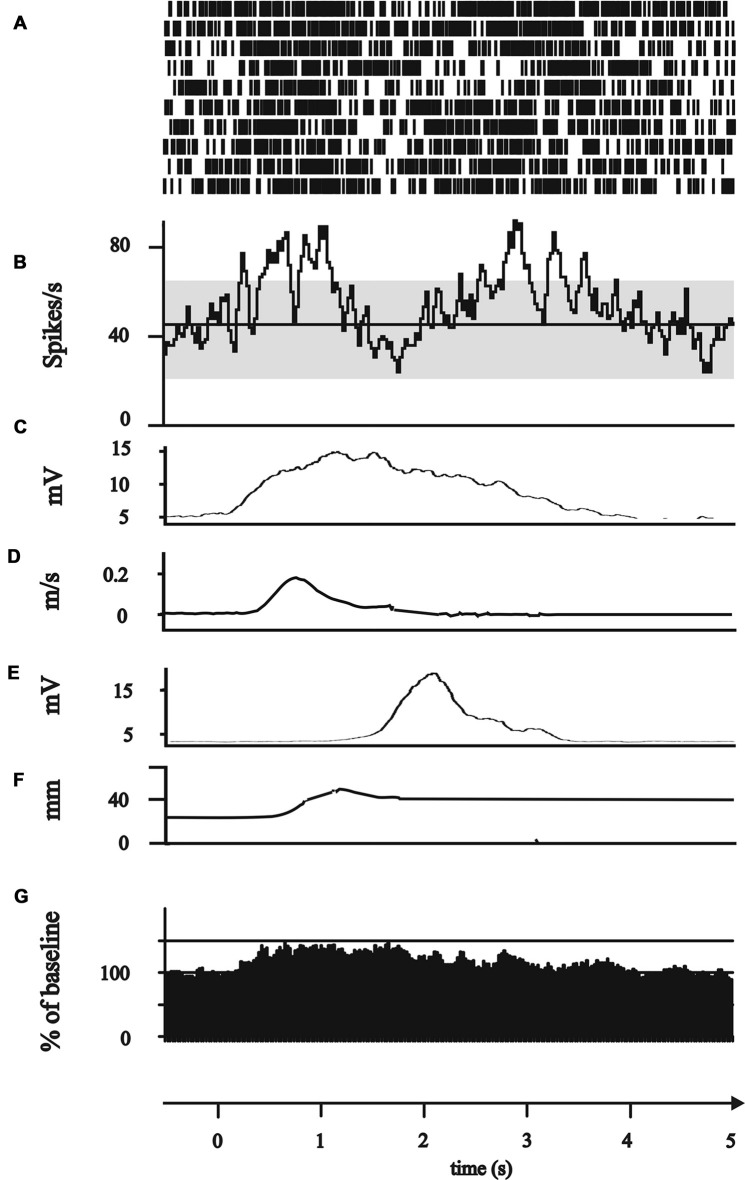
Modulation of subthalamic neuronal activity during different phases of the movement (“polymodal”). **(A–G)** All plots represent 0.5 s before to 5 s after the GO/Start signal of the movement. **(A–F)** Exemplary single neuron recorded of the lateral electrode in 1 mm above the target point. **(A)** Peri-event raster (bin 0.025 s of neuronal firing of the subthalamic cell during the 10 runs of reach-to-grasp movements. **(B)** Peri-event histogram of subthalamic activity (bin 0.025 s smoothed with boxcar filter of 3 bin) with confidence interval 95% (gray horizontal bar) of mean expected firing rate. **(C)** Rectified, smoothed and averaged EMG activity of right deltoid muscle. **(D)** Average curve of the velocity profiles of the Zebris marker attached to the hand wrist. **(E)** Rectified, smoothed and averaged EMG of the first dorsal interosseus muscle. **(F)** Profile of the distance between thumb and index finger recorded by Zebris marker. **(G)** Grand average of all 45 polymodal neurons.

The movement-associated activity of the STN neurons was investigated in more detail by calculating PSTH to different aspects of the movements, as described above (Figure 1). The movement-related activity as revealed by the PSTH was merged into four groups: (1) attentional phase before the execution of the movement; (2) The reaching phase of the reach-to-grasp movement; (3) the grip phase of the movement; (4) post-movement activation after the movement had been terminated. Not a single SUA exhibited an isolated modulation during the attentional phase. 14/114 neurons (12.28%) displayed modulation of neuronal activity only during the reaching phase (see Figure 2). 10/114 neurons (8.77%) revealed activity changes exclusively in the grip phase (see Figure 3). 6/114 neurons (5.26%) presented exclusive firing changes related to movement termination (see Figure 4). The largest proportion of STN neurons (45/114, 39.47%) exhibited polymodal activation during two or more phases of the movement (see Figure 5).

Among these polymodal neurons, 14/45 neurons (31.11%) exhibited activity changes during all phases of the movement, whereas 31/45 neurons (68.89%) showed a bimodal modulation: 10/45 neurons (22.22%) during the reach and grip phase, 10/45 neurons (22.22%) during the reaching phase and after movement termination, 11/45 neurons (24.44%) during the grip phase and after movement termination.

**Figure 6:**
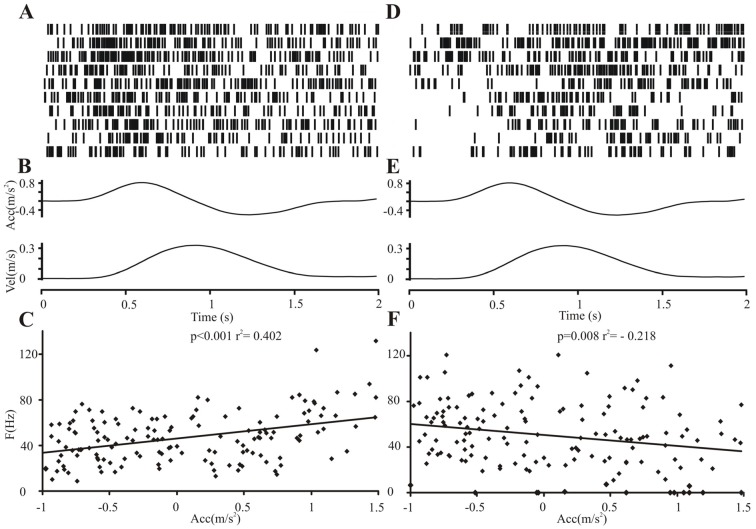
Relation of subthalamic neuronal firing activity to the kinematic velocity profile. Relation of two exemplary neighboring subthalamic neuronal firing activities of one patient to the acceleration profile during the reaching movement. Neuronal activity on the central electrode **(A–C)** was positively correlated, on the lateral electrode **(D–F)**, negatively correlated with the acceleration profile. **(A,D)** Peri-event raster (bin 0.025 s of neuronal firing of the subthalamic cell during the 10 runs of reach-to-grasp movements (2 s after GO signal). **(B,E)** Average curves of the velocity and acceleration profile during the 10 movements. **(C,F)** Plot of correlation of subthalamic single cell activity with acceleration (two-tailed Pearson, bin 100 ms).

Thus, the largest proportion of movement-related neurons (48/75, 64%) exhibited isolated or combined modulation of their spiking activity during the reaching phase of the movement. The firing of 11/14 (78.57%) “reaching” neurons was significantly correlated with the velocity or acceleration of the reaching movement (Figure 6), 6/11 (54.55%) neurons responded to velocity (3/6 [50%] positive correlations, 3/6 [50%] negative correlations) and 7/11 (63.64%) neurons responded to acceleration (2/7 [28.57%] positive correlations, 5/7 [71.42%] negative correlations). The relation to velocity or acceleration was less consistently observed in polymodal neurons. One of the 34 polymodal neurons that showed modulation during the transport phase could not be analyzed due to missing kinematic data. 14/33 (42.42%) STN polymodal neurons were significantly correlated with velocity or the acceleration during the reaching movement: 5/33 (15.15%) neurons responded to velocity (in all neurons velocity and discharge rate were modulated in the same direction); a larger fraction of polymodal neurons (9/33, 27.27%) were related to acceleration, 7/9 (77.78%) neurons were positively, 2/9 (22.22%) were negatively correlated.

**Figure 7:**
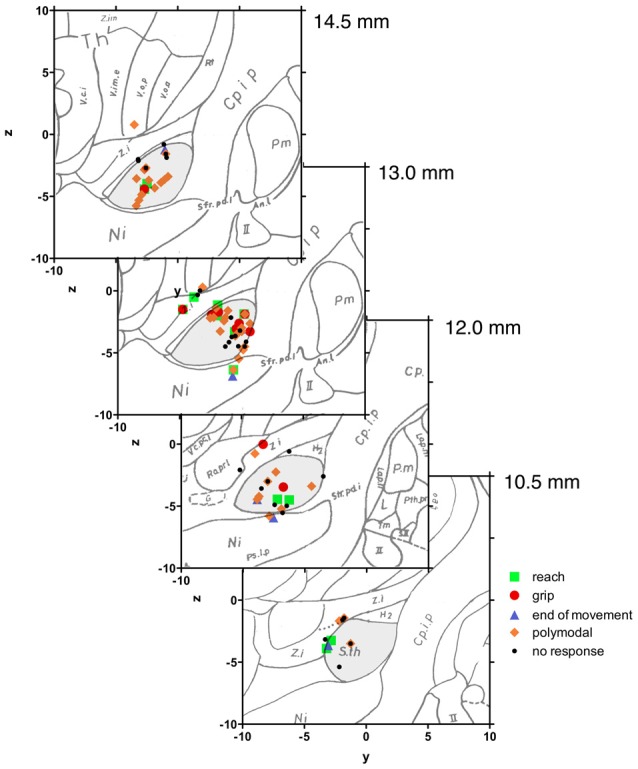
Neuroanatomical localization of recorded subthalamic neurons. Intraoperative stereotactic data were transferred to the Schaltenbrand atlas. Sagittal planes along the anterior-posterior axis from 14.5 mm to 10.5 mm. Most of the recorded sorted subthalamic neurons were localized in the dorsal part of the nucleus. There was no specific relation between the anatomical position of the subthalamic neuron and the response to movement.

We did not find a functional segregation of the different task-specific neuron types within STN, as revealed by superimposition of the recording sites onto the Schaltenbrandt stereotactic atlas (Figure 7). As expected, most of the analyzed STN neurons clustered within the dorsal part of the STN (sensorimotor region).

### Characterization of the Firing Pattern of STN Neurons

As revealed by ISIH, the firing pattern of STN neurons remained largely unaffected by the activation condition. The proportion of STN neurons exhibiting a bursting (*n* = 48 vs. 49), irregular (56 vs. 55) or tonic (10 vs. 10) firing behavior was comparable between periods of rest and movement. Neither did the CV (*p* = 0.81, *t* = 0.25) and AI (*p* = 0.37, *t* = −0.89) of ISIHs change between rest or movement, when analyzing all movement-related neurons. Only the frequency increased during movement (34.36 ± 32.73 Hz) compared to rest (31.98 ± 32.85 Hz) when all neurons were pooled (*p* ≤ 0.01, *t* = −3.67).

We conducted ANOVA testing to determine whether the resting firing properties (pattern, AI, CV, mean frequency) were associated with task responsiveness during the reach-to-grasp movement, but found no significant relationship.

### Characterization of Oscillatory Behavior

**Figure 8:**
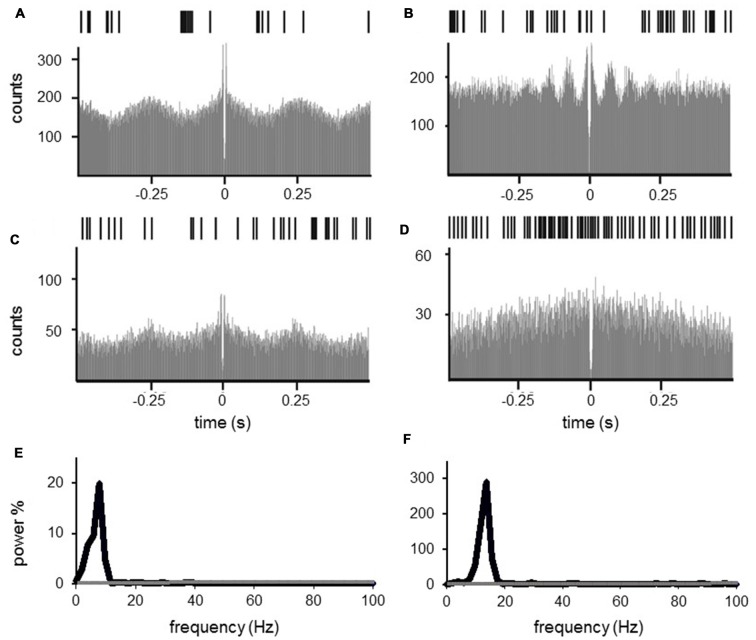
Autocorrelation of subthalamic cells. Subthalamic neuronal oscillations in the theta (1–7 Hz, **A,C,E**) and in the beta frequency (13–35 Hz, **B,D,F**) at rest **(A,B)** and during movement **(C,D)**. **(A–D)** Exemplary raster plot (upper row), 1 s and autocorrelation (lower row), −0.5 s to 0.5 s of one single unit. Grand mean powerspectrum of all single units oscillating in theta (**E**, 15 neurons) and beta frequency (**F**, 8 neurons), black line representing mean powerspectrum of all neurons at rest, gray line representing neurons during movements. The oscillating power decreased dramatically with movement.

Autocorrelation analysis (Figure 8) revealed significant oscillatory spiking behavior in 28/114 (24.6%) STN neurons at rest. Most of the neurons were oscillating in the theta (1–7 Hz) frequency (15/28 [53.6%] neurons) and in the beta (13–35 Hz) frequency (8/28 [28.6%] neurons). Only 3/28 (10.7%) neurons were oscillating in the lower and higher gamma (>36 Hz) and 2/28 (7.1%) neurons in the alpha frequency (8–12 Hz).

Most of the oscillating neurons at rest were polymodal neurons (14/28 [50%]), but there was also a large proportion of oscillating neurons that did not exhibit task-related firing changes (8/28 [28.6%]), or were classified as reaching (4/28 [14.3%]) or grip neurons (2/28 [7.1%]).

During movement, autocorrelations revealed a remarkable decrease of single neurons with significant oscillations (13.2% of neurons during movement vs. 24.6% at rest). Moreover, the spectral power of autocorrelations with significant oscillatory behavior decreased. Two-way ANOVA testing with the factors condition (rest vs. movement) and frequency (theta vs. beta frequency) for the mean power revealed a significant impact of condition (*F* = 6.121, *p* = 0.043), but not for the frequency (*F* = 0.448, *p* = 0.525) or the interaction of condition × frequency (*F* = 0.427, *p* = 0.534), indicating a reduced oscillation power during movement compared to rest irrespective of the frequencies. We did not include the gamma frequency in the ANOVA, since we only found three neurons in this frequency with low power; however, as revealed by visual inspection those gamma oscillations were also reduced and not increased by movement.

## Discussion

We found movement-related activity changes in about two-thirds of single units recorded from the dorsal STN during reach-to-grasp movements in PD patients. These neurons were responding to several kinematic aspects of the ongoing movement (polymodal neurons), most often related to the reaching phase within the reach-to-grasp movement. The reaching-related modulation of firing frequency was correlated with movement velocity or acceleration. Movement-related facilitation of neuronal activity was in general more common than inhibition. The firing frequency, but not the firing pattern, was modulated with movement onset. We found a large proportion of STN neurons exhibiting significant oscillatory firing behavior at rest, mainly in the beta and theta frequency, which diminished during movement. Most importantly, none of these task-related activity changes were observed in the premovement period, thus supporting a role of the STN in movement execution but not planning. To our knowledge, this is the first study analyzing human STN SUA in relation to different kinematic aspects of the reach-to-grasp movement.

The interpretation of our results, however, is inherently limited by the fact, that recordings were performed in patients affected by a movement disorder. Since normal control data are obviously unavailable, we cannot determine with certainty, which findings reflect physiological functions of STN and which findings relate to the pathophysiology of PD. Some insights may be derived from the comparison of our results to previous studies of movement-related basal ganglia activity in healthy, non-human primates.

Comparison of STN activity in PD patients and healthy monkeys during volitional movements revealed differences depending on the peri-movement response type. Neurons exhibiting peri-movement facilitation revealed higher neuronal discharge frequencies in Parkinsonian patients compared to healthy animals. Neuronal bursting activity with frequency-specific synchronization and diminished response variability was more often found in the Parkinsonian state interpreted as reduced information carrying capacity (Gale et al., 2009).

Task-related changes in the STN, striatum, or globus pallidus of healthy monkeys were found predominantly during the execution of proximal arm movement (DeLong et al., 1984b). A correlation of STN firing frequency with movement velocity has also been demonstrated in non-human primates during step-tracking arm movements (Georgopoulos et al., 1983; DeLong et al., 1984b). These observations underline the role of the STN in surveilling kinematic aspects of arm movements.

There are some preceding single studies in man investigating movement-associated basal ganglia activity by single cell or local field recordings in the basal ganglia. Consistent with our observations, facilitation in the STN in PD patients during voluntary arm movements was more frequently observed than movement-related inhibition as found in this study (Magariños-Ascone et al., 2000; Rodriguez-Oroz et al., 2001; Abosch et al., 2002; Levy et al., 2002; Amirnovin et al., 2004; Hanson et al., 2012). We found oscillating activity at rest in about one-quarter of investigated STN neurons, mainly in the theta and beta frequency, in accordance with previous single unit recording studies (Moran et al., 2008; Steigerwald et al., 2008) and postoperative local field potential (LFP) recordings in the dorsolateral STN (Levy et al., 2002; Kuhn et al., 2005; Weinberger et al., 2006) and GPi (Brown et al., 2001) in Parkinsonian patients. With the beginning of the reach-to-grasp movement, the oscillatory activity significantly declined comparable to previous findings of reduced oscillatory activity in single unit recordings during externally cued, voluntary, chest-to-target reaching movements (Levy et al., 2002), or beta oscillation suppression in LFP recordings prior to self- and externally-cued movements (Levy et al., 2002; Priori et al., 2002; Paradiso et al., 2003; Kuhn et al., 2004; Doyle et al., 2005; Purzner et al., 2007; Joundi et al., 2012). These findings are in accordance with the ongoing hypothesis that increased beta oscillations block the normal information flow through the basal ganglia and antagonize motor-related processing, resulting in motor impairment (Moran et al., 2008). The consistent findings during the reach-to-grasp movement investigated here, and other motor-related tasks in preceding studies, point to the hypothesis that certain neuronal characteristics seem to be “universally valid” as common integral parts of movement which are not task-specific, but a generally used neuronal code of movement.

The observation of movement-related SUA changes during the reaching phase was coherent with postoperative kinematic analyses of the reach-to-grasp movement in Parkinsonian patients with implanted STN electrodes. STN stimulation induced improvement mainly of the reaching phase, such as increment of movement velocity, but had less impact on the grip formation for object manipulation (Pötter-Nerger et al., 2013). This paradigm might represent an example for the possibility of predictive postoperative outcome by intraoperative observations.

The reach-to-grasp movement represents a precise, rapid, adaptable, goal-directed movement relying on constant recalibration in the environmental context by multistaged “feedforward” or “feedback” control (Alexander and Crutcher, 1990b; Azim and Alstermark, 2015). The initial variable motor command is corrected and adapted by afferent visual and proprioceptive feedback and internal, centrally-generated copies of the motor command. Within this “feedforward-feedback” system during forelimb movements, the role of the STN is still a matter of debate (Alexander and Crutcher, 1990b). Recent studies revealed evidence for the involvement of the STN in the “feedforward” control by observations of a single unit (Amirnovin et al., 2004) and LFP changes prior to movement (Kuhn et al., 2004; Williams et al., 2005), as well as findings during motor imagery (Kuhn et al., 2006) in the STN. On the other hand, there is evidence for afferent “feedback” processing of the ongoing motor program in the animal model and in humans. There were late increases of neuronal discharge rates with or even after EMG onset of the major portion of neurons in STN microrecordings (DeLong et al., 1984b; Wichmann et al., 1994a; Cheruel et al., 1996) and late beta synchronization after successful completion of the movement (Kuhn et al., 2004, 2006). In our observations, we found no activity changes prior to movement during the attentional phase, but mainly during movement or after the actual movement had been finished. We therefore suggest that in this specific experimental setting of a more or less “stereotyped” motor task, the STN was more involved in the “feedback control” of the movement than in the “feedforward” motor planning.

## Author Contributions

MP-N: development of the experimental set up, conduct of experiments, data analyses, manuscript writing. RR: support during conduct of experiments, data analyses, manuscript writing. FS: conduct of experiments, data analyses, manuscript writing. JAH: data analyses, manuscript writing. JH: conduct of the experiments, manuscript writing. CKEM: manuscript writing. WH: manuscript writing. UR-P: support data analyses, manuscript writing. DF: support during conduct of the experiments, manuscript writing. MM: support during conduct of the experiments, manuscript writing. CG: manuscript writing. GD: advice on data analyses, manuscript writing. JV: development of the experimental idea and set up, conduct of experiments, data analyses, manuscript writing.

## Conflict of Interest Statement

MP-N received lecture fees from St. Jude, grants from Medtronic, study fees from Boston Scientific, and has been serving as consultant for Abbvie and Licher. RR received travel grants from Medtronic and Abbvie. FS received speaker and consultant honoraria from Boston Scientific, St. Jude Medical and performed research projects sponsored by Boston Scientific and Medtronic. CKEM served as consultant for Alpha Omega, St. Jude and Brainlab. WH received lecture fees, honoraria for serving on advisory boards, and travel grants from Boston Scientific, Medtronic Inc., and St. Jude Medical, Inc. DF has received lecture fees from Medtronic. MM has served as a consultant for Medtronic, Philips Healthcare, B Braun and Boston Scientific and has received honoraria from these companies. GD has received lecture fees from Almirall and Novartis and has been serving as a consultant for Boston Scientific. He received royalties from Thieme publishers. He is a government employee and receives through his institution funding for his research from the German Research Council, the German Ministry of Education and Health and Medtronic. JV has served as a consultant for Boston Scientific and Medtronic. He received honoraria from Allergan, Merz; UCB, Abbvie, TEVA, Zambon, Bial. The other authors declare that the research was conducted in the absence of any commercial or financial relationships that could be construed as a potential conflict of interest.

## References

[B1] Abosch A. Hutchison W. D. Saint-Cyr J. A. Dostrovsky J. O. Lozano A. M. (2002). Movement-related neurons of the subthalamic nucleus in patients with Parkinson disease. J. Neurosurg. 97, 1167–1172. 10.3171/jns.2002.97.5.1167 12450039

[B2] Aflalo T. Kellis S. Klaes C. Lee B. Shi Y. Pejsa K.. (2015). Neurophysiology. Decoding motor imagery from the posterior parietal cortex of a tetraplegic human. Science 348, 906–910. 10.1126/science.aaa5417 25999506 PMC4896830

[B3] Alberts J. L. Saling M. Adler C. H. Stelmach G. E. (2000). Disruptions in the reach-to-grasp actions of Parkinson’s patients. Exp. Brain Res. 134, 353–362. 10.1007/s002210000468 11045360

[B4] Alexander G. E. Crutcher M. D. (1990a). Neural representations of the target (goal) of visually guided arm movements in three motor areas of the monkey. J. Neurophysiol. 64, 164–178. 2388063 10.1152/jn.1990.64.1.164

[B5] Alexander G. E. Crutcher M. D. (1990b). Functional architecture of basal ganglia circuits: neural substrates of parallel processing. Trends Neurosci. 13, 266–271. 10.1016/0166-2236(90)90107-l 1695401

[B6] Amirnovin R. Williams Z. M. Cosgrove G. R. Eskandar E. N. (2004). Visually guided movements suppress subthalamic oscillations in Parkinson’s disease patients. J. Neurosci. 24, 11302–11306. 10.1523/JNEUROSCI.3242-04.2004 15601936 PMC6730370

[B7] Azim E. Alstermark B. (2015). Skilled forelimb movements and internal copy motor circuits. Curr. Opin. Neurobiol. 33, 16–24. 10.1016/j.conb.2014.12.009 25588912 PMC4497943

[B9] Bergman H. Wichmann T. Karmon B. DeLong M. R. (1994). The primate subthalamic nucleus. II. Neuronal activity in the MPTP model of parkinsonism. J. Neurophysiol. 72, 507–520. 7983515 10.1152/jn.1994.72.2.507

[B11] Brown P. Oliviero A. Mazzone P. Insola A. Tonali P. Di Lazzaro V. (2001). Dopamine dependency of oscillations between subthalamic nucleus and pallidum in Parkinson’s disease. J. Neurosci. 21, 1033–1038. 11157088 10.1523/JNEUROSCI.21-03-01033.2001 PMC6762327

[B12] Cavina-Pratesi C. Monaco S. Fattori P. Galletti C. McAdam T. D. Quinlan D. J.. (2010). Functional magnetic resonance imaging reveals the neural substrates of arm transport and grip formation in reach-to-grasp actions in humans. J. Neurosci. 30, 10306–10323. 10.1523/JNEUROSCI.2023-10.2010 20685975 PMC6634677

[B13] Cheruel F. Dormont J. F. Farin D. (1996). Activity of neurons of the subthalamic nucleus in relation to motor performance in the cat. Exp. Brain Res. 108, 206–220. 10.1007/bf00228095 8815030

[B15] DeLong M. R. Alexander G. E. Georgopoulos A. P. Crutcher M. D. Mitchell S. J. Richardson R. T. (1984a). Role of basal ganglia in limb movements. Hum. Neurobiol. 2, 235–244. 6715208

[B16] DeLong M. R. Georgopoulos A. P. Crutcher M. D. Mitchell S. J. Richardson R. T. Alexander G. E. (1984b). Functional organization of the basal ganglia: contributions of single-cell recording studies. Ciba Found. Symp. 107, 64–82. 10.1002/9780470720882.ch5 6389041

[B17] Doyle L. M. Kuhn A. A. Hariz M. Kupsch A. Schneider G. H. Brown P. (2005). Levodopa-induced modulation of subthalamic beta oscillations during self-paced movements in patients with Parkinson’s disease. Eur. J. Neurosci. 21, 1403–1412. 10.1111/j.1460-9568.2005.03969.x 15813950

[B18] Fattori P. Raos V. Breveglieri R. Bosco A. Marzocchi N. Galletti C. (2010). The dorsomedial pathway is not just for reaching: grasping neurons in the medial parieto-occipital cortex of the macaque monkey. J. Neurosci. 30, 342–349. 10.1523/JNEUROSCI.3800-09.2010 20053915 PMC6632536

[B20] Gale J. T. Shields D. C. Jain F. A. Amirnovin R. Eskandar E. N. (2009). Subthalamic nucleus discharge patterns during movement in the normal monkey and Parkinsonian patient. Brain Res. 1260, 15–23. 10.1016/j.brainres.2008.12.062 19167367 PMC2819925

[B21] Georgopoulos A. P. DeLong M. R. Crutcher M. D. (1983). Relations between parameters of step-tracking movements and single cell discharge in the globus pallidus and subthalamic nucleus of the behaving monkey. J. Neurosci. 3, 1586–1598. 6875658 10.1523/JNEUROSCI.03-08-01586.1983 PMC6564524

[B22] Gutteling T. P. Petridou N. Dumoulin S. O. Harvey B. M. Aarnoutse E. J. Kenemans J. L.. (2015). Action preparation shapes processing in early visual cortex. J. Neurosci. 35, 6472–6480. 10.1523/JNEUROSCI.1358-14.2015 25904798 PMC6605225

[B23] Hanson T. L. Fuller A. M. Lebedev M. A. Turner D. A. Nicolelis M. A. (2012). Subcortical neuronal ensembles: an analysis of motor task association, tremor, oscillations and synchrony in human patients. J. Neurosci. 32, 8620–8632. 10.1523/JNEUROSCI.0750-12.2012 22723703 PMC3502028

[B24] Hassani O. K. Mouroux M. Féger J. (1996). Increased subthalamic neuronal activity after nigral dopaminergic lesion independent of disinhibition via the globus pallidus. Neuroscience 72, 105–115. 10.1016/0306-4522(95)00535-8 8730710

[B25] Hutchison W. D. Allan R. J. Opitz H. Levy R. Dostrovsky J. O. Lang A. E.. (1998). Neurophysiological identification of the subthalamic nucleus in surgery for Parkinson’s disease. Ann. Neurol. 44, 622–628. 10.1002/ana.410440407 9778260

[B26] Jaeger D. Gilman S. Aldridge J. W. (1995). Neuronal activity in the striatum and pallidum of primates related to the execution of externally cued reaching movements. Brain Res. 694, 111–127. 10.1016/0006-8993(95)00780-t 8974634

[B27] Jeannerod M. (1984). The timing of natural prehension movements. J. Mot. Behav. 16, 235–254. 10.1080/00222895.1984.10735319 15151851

[B28] Joundi R. A. Brittain J. S. Green A. L. Aziz T. Z. Brown P. Jenkinson N. (2012). Oscillatory activity in the subthalamic nucleus during arm reaching in Parkinson’s disease. Exp. Neurol. 236, 319–326. 10.1016/j.expneurol.2012.05.013 22634757

[B29] Klaes C. Kellis S. Aflalo T. Lee B. Pejsa K. Shanfield K.. (2015). Hand shape representations in the human posterior parietal cortex. J. Neurosci. 35, 15466–15476. 10.1523/JNEUROSCI.2747-15.2015 26586832 PMC4649012

[B30] Kuhn A. A. Doyle L. Pogosyan A. Yarrow K. Kupsch A. Schneider G. H.. (2006). Modulation of beta oscillations in the subthalamic area during motor imagery in Parkinson’s disease. Brain 129, 695–706. 10.1093/brain/awh715 16364953

[B31] Kuhn A. A. Trottenberg T. Kivi A. Kupsch A. Schneider G. H. Brown P. (2005). The relationship between local field potential and neuronal discharge in the subthalamic nucleus of patients with Parkinson’s disease. Exp. Neurol. 194, 212–220. 10.1016/j.expneurol.2005.02.010 15899258

[B32] Kuhn A. A. Williams D. Kupsch A. Limousin P. Hariz M. Schneider G. H.. (2004). Event-related beta desynchronization in human subthalamic nucleus correlates with motor performance. Brain 127, 735–746. 10.1093/brain/awh106 14960502

[B33] Lebedev M. A. Nelson R. J. (1995). Rhythmically firing (20–50 Hz) neurons in monkey primary somatosensory cortex: activity patterns during initiation of vibratory-cued hand movements. J. Comput. Neurosci. 2, 313–334. 10.1007/bf00961443 8746405

[B34] Lebedev M. A. Nelson R. J. (1999). Rhythmically firing neostriatal neurons in monkey: activity patterns during reaction-time hand movements. J. Neurophysiol. 82, 1832–1842. 10515972 10.1152/jn.1999.82.4.1832

[B35] Lebedev M. A. Wise S. P. (2000). Oscillations in the premotor cortex: single-unit activity from awake, behaving monkeys. Exp. Brain Res. 130, 195–215. 10.1007/s002210050022 10672473

[B36] Lehmann S. J. Scherberger H. (2015). Spatial representations in local field potential activity of primate anterior intraparietal cortex (AIP). PLoS One 10:e0142679. 10.1371/journal.pone.0142679 26554592 PMC4640530

[B37] Levy R. Ashby P. Hutchison W. D. Lang A. E. Lozano A. M. Dostrovsky J. O. (2002). Dependence of subthalamic nucleus oscillations on movement and dopamine in Parkinson’s disease. Brain 125, 1196–1209. 10.1093/brain/awf128 12023310

[B39] Magariños-Ascone C. M. Figueiras-Mendez R. Riva-Meana C. Córdoba-Fernández A. (2000). Subthalamic neuron activity related to tremor and movement in Parkinson’s disease. Eur. J. Neurosci. 12, 2597–2607. 10.1046/j.1460-9568.2000.00127.x 10947834

[B40] Mason C. R. Hendrix C. M. Ebner T. J. (2006). Purkinje cells signal hand shape and grasp force during reach-to-grasp in the monkey. J. Neurophysiol. 95, 144–158. 10.1152/jn.00492.2005 16162833

[B41] Michaels J. A. Dann B. Intveld R. W. Scherberger H. (2015). Predicting reaction time from the neural state space of the premotor and parietal grasping network. J. Neurosci. 35, 11415–11432. 10.1523/JNEUROSCI.1714-15.2015 26269647 PMC6605125

[B42] Monaco S. Sedda A. Cavina-Pratesi C. Culham J. C. (2015). Neural correlates of object size and object location during grasping actions. Eur. J. Neurosci. 41, 454–465. 10.1111/ejn.12786 25400211

[B43] Moran A. Bergman H. Israel Z. Bar-Gad I. (2008). Subthalamic nucleus functional organization revealed by parkinsonian neuronal oscillations and synchrony. Brain 131, 3395–3409. 10.1093/brain/awn270 18986993

[B44] Paradiso G. Saint-Cyr J. A. Lozano A. M. Lang A. E. Chen R. (2003). Involvement of the human subthalamic nucleus in movement preparation. Neurology 61, 1538–1545. 10.1212/01.WNL.0000096021.28967.57 14663039

[B45] Pötter-Nerger M. Habben A. Herzog J. Falk D. Mehdorn M. H. Deuschl G.. (2013). Kinematic effects of subthalamic stimulation on reach-to-grasp movements in Parkinson’s disease. Parkinsonism Relat. Disord. 19, 32–36. 10.1016/j.parkreldis.2012.06.018 22795308

[B46] Priori A. Foffani G. Pesenti A. Bianchi A. Chiesa V. Baselli G.. (2002). Movement-related modulation of neural activity in human basal ganglia and its L-DOPA dependency: recordings from deep brain stimulation electrodes in patients with Parkinson’s disease. Neurol. Sci. 23, S101–S102. 10.1007/s100720200089 12548363

[B47] Pruszynski J. A. Diedrichsen J. (2015). Neuroscience. Reading the mind to move the body. Science 348, 860–861. 10.1126/science.aab3464 25999491

[B48] Purzner J. Paradiso G. O. Cunic D. Saint-Cyr J. A. Hoque T. Lozano A. M.. (2007). Involvement of the basal ganglia and cerebellar motor pathways in the preparation of self-initiated and externally triggered movements in humans. J. Neurosci. 27, 6029–6036. 10.1523/JNEUROSCI.5441-06.2007 17537974 PMC6672263

[B49] Raz A. Feingold A. Zelanskaya V. Vaadia E. Bergman H. (1996). Neuronal synchronization of tonically active neurons in the striatum of normal and parkinsonian primates. J. Neurophysiol. 76, 2083–2088. 8890317 10.1152/jn.1996.76.3.2083

[B50] Rodriguez-Oroz M. C. Rodriguez M. Guridi J. Mewes K. Chockkman V. Vitek J.. (2001). The subthalamic nucleus in Parkinson’s disease: somatotopic organization and physiological characteristics. Brain 124, 1777–1790. 10.1093/brain/124.9.1777 11522580

[B51] Romanelli P. Bronte-Stewart H. Heit G. Schaal D. W. Esposito V. (2004). The functional organization of the sensorimotor region of the subthalamic nucleus. Stereotact. Funct. Neurosurg. 82, 222–229. 10.1159/000082778 15604597

[B52] Romanelli P. Esposito V. Schaal D. W. Heit G. (2005). Somatotopy in the basal ganglia: experimental and clinical evidence for segregated sensorimotor channels. Brain Res. Rev. 48, 112–128. 10.1016/j.brainresrev.2004.09.008 15708631

[B53] Steigerwald F. Hinz L. Pinsker M. O. Herzog J. Stiller R. U. Kopper F.. (2005). Effect of propofol anesthesia on pallidal neuronal discharges in generalized dystonia. Neurosci. Lett. 386, 156–159. 10.1016/j.neulet.2005.06.012 16024174

[B54] Steigerwald F. Pötter M. Herzog J. Pinsker M. Kopper F. Mehdorn H.. (2008). Neuronal activity of the human subthalamic nucleus in the parkinsonian and nonparkinsonian state. J. Neurophysiol. 100, 2515–2524. 10.1152/jn.90574.2008 18701754

[B55] Theodosopoulos P. V. Marks W. J. Jr. Christine C. Starr P. A. (2003). Locations of movement-related cells in the human subthalamic nucleus in Parkinson’s disease. Mov. Disord. 18, 791–798. 10.1002/mds.10446 12815658

[B56] van Kan P. L. McCurdy M. L. (2002). Discharge of primate magnocellular red nucleus neurons during reaching to grasp in different spatial locations. Exp. Brain Res. 142, 151–157. 10.1007/s00221-001-0924-5 11797092

[B57] Weinberger M. Mahant N. Hutchison W. D. Lozano A. M. Moro E. Hodaie M.. (2006). Beta oscillatory activity in the subthalamic nucleus and its relation to dopaminergic response in Parkinson’s disease. J. Neurophysiol. 96, 3248–3256. 10.1152/jn.00697.2006 17005611

[B58] Wenger K. K. Musch K. L. Mink J. W. (1999). Impaired reaching and grasping after focal inactivation of globus pallidus pars interna in the monkey. J. Neurophysiol. 82, 2049–2060. 10561386 10.1152/jn.1999.82.5.2049

[B59] Wichmann T. Bergman H. Delong M. R. (1994a). The primate subthalamic nucleus. I. Functional properties in intact animals. J. Neurophysiol. 72, 494–506. 7983514 10.1152/jn.1994.72.2.494

[B60] Wichmann T. Bergman H. DeLong M. R. (1994b). The primate subthalamic nucleus. III. Changes in motor behavior and neuronal activity in the internal pallidum induced by subthalamic inactivation in the MPTP model of parkinsonism. J. Neurophysiol. 72, 521–530. 7983516 10.1152/jn.1994.72.2.521

[B61] Williams D. Kühn A. Kupsch A. Tijssen M. van Bruggen G. Speelman H.. (2005). The relationship between oscillatory activity and motor reaction time in the parkinsonian subthalamic nucleus. Eur. J. Neurosci. 21, 249–258. 10.1111/j.1460-9568.2004.03817.x 15654862

